# Education and Protein Supplementation Improve Nutritional Biomarkers among Hypoalbuminemic Peritoneal Dialysis Patients: A Quasi-Experimental Design

**DOI:** 10.3390/healthcare7040135

**Published:** 2019-11-05

**Authors:** Tuyen Van Duong, Chang-An Tsao, Evelyn Yang, Ching-Hsiu Peng, Yi-Cheng Hou, Yan-Chen Su, Jui-Ting Chang, Shwu-Huey Yang

**Affiliations:** 1 School of Nutrition and Health Sciences, Taipei Medical University, Taipei 110, Taiwan; duongtuyenvna@gmail.com (T.V.D.); poll_linda@yahoo.com.tw (C.-A.T.); anny321@tmu.edu.tw (Y.-C.H.); 2 Department of Nutrition Therapy, Dalin Tzu Chi Hospital, Buddhist Tzu Chi Medical Foundation, Chiayi 622, Taiwan; 3 Department of Obstetrics and Gynecology, Chung Shan Medical University Hospital, Taichung City 402, Taiwan; evelyn.yang@hotmail.com; 4 Department of Nephrology, Taipei Tzu Chi Hospital, Buddhist Tzu Chi Medical Foundation, Taipei 231, Taiwan; soranos2008@yahoo.com.tw; 5 Department of Nutrition, Taipei Tzu Chi Hospital, Buddhist Tzu Chi Medical Foundation, Taipei 231, Taiwan; 6 Department of Nutrition, Shin Kong Wu Ho-Su Memorial Hospital, Taipei 111, Taiwan; bedmoon1982@gmail.com; 7 Department of Nephrology, Shin Kong Wu Ho-Su Memorial Hospital, Taipei 111, Taiwan; m007454@ms.skh.org.tw; 8 Research Center of Geriatric Nutrition, Taipei Medical University, Taipei 110, Taiwan; 9 Nutrition Research Center, Taipei Medical University Hospital, Taipei 110, Taiwan

**Keywords:** nutritional education, protein supplements, milk protein, soy protein, peritoneal dialysis, biomarkers, hypoalbuminemia, serum albumin, serum phosphorus, experimental study

## Abstract

Protein-energy wasting is prevalent in peritoneal dialysis patients, which causes a heavy burden for individuals and healthcare systems. We aimed to investigate the effect of nutritional education, and/or protein supplementation on nutritional biomarkers in hypoalbuminemic peritoneal dialysis patients. A quasi-experimental study was conducted in two dialysis centers at Taipei Tzu Chi Hospital and Shin Kong Wu Ho-Su Memorial Hospital. Patients were allocated in three groups including control (*n* = 12), milk protein (*n* = 21) and soy protein (*n* = 20). All patients received dietary guidelines from dietitians and completed 3-day dietary records during monthly visits for consecutive three months. Nutrients were analyzed using Nutritionist Professional software. Blood urea nitrogen (BUN), creatinine, albumin, total protein, hemoglobin, serum calcium, phosphorus, sodium, and potassium were assessed monthly. Total cholesterol and triglycerides were measured every three months. After three-month intervention, protein intake (percent of total calories), and serum albumin were significantly increased in three groups. Protein, phosphorus intake, and BUN were increased in two intervention groups. Total serum protein increased in control and milk protein groups, and creatinine increased the control group. Serum phosphorus was not significantly changed. Nutritional education alone, or combined with protein supplementation, significantly improve protein intake, and nutritional status by increasing serum albumin, but not serum phosphorus in hypoalbuminemic peritoneal dialysis patients.

## 1. Introduction

Globally, more than 270,000 patients undergoing peritoneal dialysis were reported in 2015 [1]. The annual growth rate of peritoneal dialysis was approximately 8%, exceeding that of hemodialysis (6–7%) [1]. The number of peritoneal dialysis patients in the Asia-Pacific region is significantly increasing each year [1,2]. This happened because peritoneal dialysis is more cost-effective than hemodialysis, which gives patients more freedom over their lives [1,3]. In addition, the governments allow reimbursement of peritoneal dialysis but not hemodialysis. Moreover, it is comparable in outcome and quality between two therapies [2]. The etiology of protein-energy wasting (PEW) in patients treated with either peritoneal or hemodialysis is comparable, and the prevalence of PEW is relatively high, ranging from 18% to 56% [4,5,6,7]. It is associated with a higher risk for morbidity and mortality [8]. Therefore, it is important to prevent and treat PEW in end stage renal disease (ESRD) patients [5,9,10].

Poor dietary intake, especially low protein intake, is a major cause of PEW in peritoneal dialysis patients [11,12]. In addition, a number of other factors contribute to PEW development such as loss of residual kidney function, inadequate dialysis, uremia on protein synthesis, inflammation, loss of protein, and amine-based acids during dialysis [7,9,13,14,15,16,17]. These further exacerbate poor dietary intake [16]. Peritoneal dialysis patients meanwhile need adequate protein intake to compromise negative nitrogen balance [18,19].

Nutritional therapy is an integrated approach that should be early implemented to improve clinical outcomes [5,9,19,20]. Protein supplementation is an effective intervention in improving PEW status in chronic kidney disease (CKD) patients [5,21]. In addition, nutritional education contributes to improving diet quality [22]. Dietary counseling is one of the important approaches to manage PEW in peritoneal dialysis patients [7]. Therefore, we aimed to investigate the effect of nutritional education and/or oral protein supplements on nutritional biomarkers in peritoneal dialysis patients with hypoalbuminemia.

## 2. Methods

### 2.1. Study Design, Settings, and Patients

A quasi-experimental study was conducted over a three-month period. The study was conducted in two peritoneal dialysis centers at Taipei Tzu Chi Hospital (from September 2015 to December 2016) and Shin Kong Wu Ho-Su Memorial Hospital (from October 2017 to February 2018).

**Figure 1:**
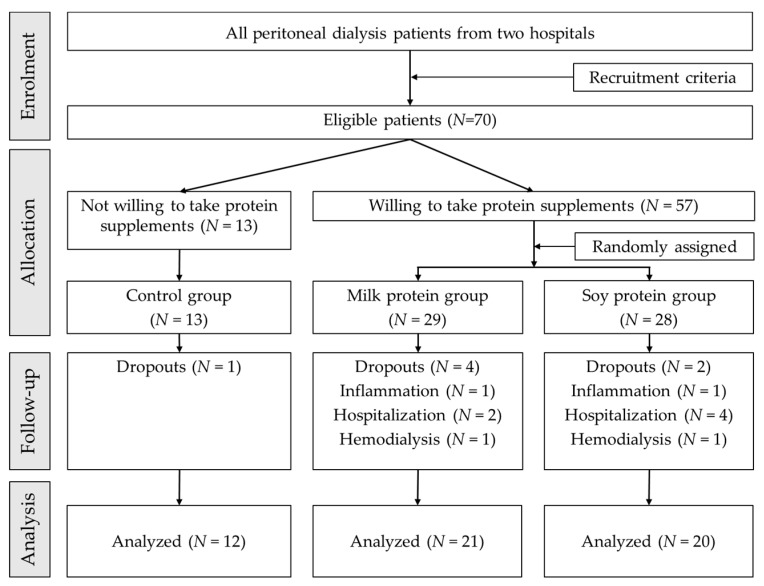
Study flowchart.

Patients recruited were those who classified as hypoalbuminemia with serum albumin <3.8 g/dL. Hypoalbuminemia was considered as biochemical criteria in diagnosis of PEW, defined by the International Society of Renal Nutrition and Metabolism [23]. In addition, patients included were those had regular peritoneal treatment for at least three months, aged 25 to 85 years, had no intravenous nutrition therapy or albumin injection, with stable clinical symptoms and no unexpected renal transplantation in the short term or other surgical procedures, and were able to complete the experiment. Patients excluded from the study were those who had all-day tube perfusion or acute inflammatory infections, or hospitalized, or liver failure or malignant tumors, or adding amino acid dialysate to their regimen. A total sample of 70 eligible patients was recruited (Figure 1). The checklist was completed and presented in Table S1, according to CONSORT guidelines [24,25].

### 2.2. Interventions

Patients were divided into three groups depending on their willingness to take protein supplements. Patients who did not intend to take the supplements were assigned to the control group and those willing to take regular protein supplements were assigned randomly to the milk protein intake group or the soy protein intake group.

At each monthly visit, a registered dietitian checked the 3-day dietary records of patients and their dietary compliances. The same dietitian then provided nutritional education on any topics in which patients were deficient.

After a nutritional education session on the day of the peritoneal dialysis clinic, patients in the control group consumed routine foods of their choices. Patients in the intervention groups (in the milk protein and the soy protein groups) were given one-month supply of daily protein supplements. The milk protein product named S-P93^®^, Sentosa Corporation Limited, Taipei, Taiwan. The soy protein product named Nu-Reno^®^; Nutritec-enjoy Corporation Limited, Taipei, Taiwan. Patients were instructed to daily drink the protein supplements with the same amount, to replace about two servings of protein. The nutritional ingredients contained in the protein supplements are shown in Table 1.

At the clinic visit, a dietitian gave milk or soy protein supplements to patients and checked the previous package to evaluate the adherence. Patients were interviewed by the same dietician using the 24 h dietary recall to assess the dietary intake and supplement intake. Seventy patients who met the screening criteria and agreed to participate were initially recruited. After signing the consent forms, patients were enrolled in the three-month intervention.

### 2.3. Dietary Intake Assessment

Patients were instructed by dietitians to complete three-day dietary record using the 24-h dietary recall form (one dialysis weekday, one non-dialysis weekday, one non-dialysis weekend day). The nutrients were analyzed using Nutritionist Professional software (E-Kitchen Business, Taipei, Taiwan).

### 2.4. Clinical and Biochemical Parameters

The information related to gender, body mass index, types of peritoneal dialysis, medical history, urea clearance test, residual renal function (RRF), and normalized protein catabolic ratio (nPCR) were collected using chart review.

Biochemical parameters were collected from hospital laboratories including blood urea nitrogen (BUN), Albumin (Alb), total protein (TP), Creatinine (Cre), Calcium (Ca), Hemoglobin (Hb), Potassium (K), Sodium (Na), Phosphorous (P), and total dialysate volume. These parameters were measured at baseline, month 1, month 2, and month 3. Total cholesterol (TC), Triglyceride (TG) were assessed at baseline and month 3.

Albumin (Alb) was measured by bromocresol purple (BCP) at Taipei Tzu Chi Hospital, bromocresol green (BCG) at Shin Kong Wu Ho-Su Memorial Hospital. The Alb values were converted using the following formula: Alb (BCG) = 5.5 mg/dL + Alb (BCP) for statistical analysis.

Taipei Tzu Chi Hospital measured total serum calcium, while Shin Kong Wu Ho-Su Memorial Hospital measured free serum calcium. The information provided by Shin Kong Wu Ho-Su Memorial Hospital Department indicated that free serum calcium accounted for 45% of total serum calcium. Total serum calcium was used for statistical analysis.

### 2.5. Ethical Approval

The study was approved by Institutional Review Boards (IRB No.: 04-XD05-030, and IRB No.: 20170302R). Patients provided written consent before their participation.

### 2.6. Statistical Analysis

The descriptive analysis was conducted to explore the distribution of studied variables. Mean ± standard deviation (SD) or frequency were presented. The Shapiro–Wilk normal distribution test was used to examine the normality of continuous variables. The paired *t*-test, one-way ANOVA test, one-way repeated measure ANOVA and Fisher’s least significant difference test, the Mann–Whitney U test, the Wilcoxon signed-rank test, and the chi-square test were utilized appropriately to examine the effects. Data were analyzed using the statistical software SPSS version 22.0 (IBM Corp, Armonk, NY, USA). The *p*-value < 0.05 was considered statistically significant.

## 3. Results 

### 3.1. Patients’ Characteristics

During the study period, one patient dropped out in the control group; four patients dropped out, one infected, two hospitalized, and one switched to hemodialysis in milk protein group; two patients dropped out, one had infection, four hospitalized, and one switched to hemodialysis in the soy protein group. Finally, a total of 53 patients completed the study and were included in the data analysis: 12 patients in the control group, 21 patients in the milk protein group, and 20 patients in the soy protein group (Figure 1).

Patients’ characteristics at baseline were not significantly differed among three studied groups which were presented in Table 2.

### 3.2. Effects on Daily Nutrient Intake

At the baseline, nutrients’ intake was not significantly different among three studied groups. After three months, the protein intake (g/kg) in the milk protein group was significantly higher than in the control group (*p* = 0.017). The dietary phosphorus intake (mg) in both intervention groups (the milk protein group and the soy protein group) was significantly higher than that in the control group with *p* = 0.010 and *p* = 0.036, respectively. The dietary calcium intake (mg) in the milk group was significantly higher than that in the soy protein group (*p* = 0.008) and the control group (*p* < 0.001; Table 3).

In all groups, the protein percent of total calories (%) was significantly increased after the intervention. The milk protein group and the soy protein group also had significantly increased absolute protein intake (g/kg) and dietary phosphorus intake (mg) in month three (*p* < 0.05; Table 3). The milk protein group had a significant increase in dietary calcium (mg) after the intervention (*p* < 0.05; Table 3).

### 3.3. Effects on Biochemical Parameters

The biochemical values in the three groups did not differ at baseline. In three groups, albumin levels after the three-month intervention was significantly higher than that at baseline (*p* < 0.05). In the milk protein and soy protein groups, BUN levels were significantly increased as compared to that at baseline, and the increase in the intervention groups was higher than that in the control group (*p* < 0.05). Serum potassium was significantly increased in the milk protein group (*p* < 0.05). In the control group, the serum creatinine, total protein, and phosphorus were significantly higher than at baseline (*p* < 0.05; Table 4).

## 4. Discussion

At the baseline, the average calorie and protein intake were lower than the amounts recommended in the National Kidney Foundation Kidney Disease Outcomes Quality Initiative (NKF/KDOQI) guidelines [18]. Patients treated with peritoneal dialysis have difficulties with achieving their nutritional requirements due to insufficient food intake, which might be one of the reasons for PEW. It is also mentioned previously that poor dietary intake is common in patients on peritoneal dialysis [12,16]. In addition, previous studies conducted in Korea and Hong Kong also demonstrate that consumptions of energy and protein are lower than the recommendations in peritoneal dialysis patients [26,27].

After three months of intervention, the intake of different nutrients was improved, and significantly increased in protein intake in all groups of patients. The intake of protein has reached the recommendation level in intervention groups. Oral protein supplementation (Otsuka Nutrition Pharmaceutical (ONCE) dialyze formula (ODF)) was found to increase the calorie and protein intake in hypoalbuminemic peritoneal dialysis patients [28]. Another study conducted by Sahathevan and colleagues in Malaysia found that oral protein supplementation was significantly associated with increased protein intake in an intervention group, and increased calorie intake in control groups [11]. Our study showed that calorie intake of patients in the three groups did not change significantly after three months of nutritional intervention. A previous study conducted in Australia also illustrates no significant increased calorie intake after 16 weeks’ oral nutritional supplementation [29]. This may be due to the low protein energy (about 200 kcal/day) from products in this study (Table 1). In addition, patients with peritoneal dialysis are often trapped in a vicious cycle of poor appetite and PEW, and nutrition and health education during the trial did not result in a significant increase in energy intake. Long-term trials and follow-up are highly suggested in future studies.

The significantly higher protein and phosphorus intake were found in the current study. Since high protein intake is linked to high phosphorus intake, it is a challenge to control blood phosphorus levels for dialysis patients while they are required to have an increased protein diet. In addition, after three-month intervention, the serum phosphorus level was significantly increased in control groups, but not in the intervention groups. This could be explained by the fact that, when patients were advised to have more protein intake, they might choose foods rich in both protein and phosphorus, whereas, in the groups with supplements, the amount of phosphorus was more controlled. Therefore, replacing dietary protein with 30–40 g of low-phosphorus and low-potassium whey protein in dialysis patients with hyperphosphatemia significantly reduces the phosphorus intake and serum phosphorus levels of patients after three months [30]. A meta-analysis of 12 clinical trials in renal failure patients elucidates that consumption of soy protein is significantly associated with lowering the concentration of serum phosphorus, as compared with consumption of animal protein [31].

In our study, after three-month nutritional education and protein supplementation, albumin was found to be increased in all groups, total protein was increased in control and milk protein groups, while BUN was increased in two intervention groups. Results of a previous study demonstrated that oral protein supplements (dietary renal-specific formula) could improve pre-albumin, BUN, and body weight in hypoalbuminemic patients [28]. Other types of oral protein supplements like whey protein could also improve nutritional status (serum albumin, normalized protein equivalent of total nitrogen appearance, and lean tissue index) in peritoneal dialysis patients [32]. In addition, it was summarized from 15 randomized clinical trials that the serum albumin level increased by energy, or protein/amino acid supplements in dialysis patients [33]. A study conducted on malnourished peritoneal patients by Sahathevan and colleagues in Malaysia showed that whey protein supplementation significantly increased total protein in both intervention groups, while it increased serum albumin in the control group [11]. In addition, another study conducted by Dong and colleagues in China found that higher daily protein intake in intervention group was associated with higher serum albumin in patients treated peritoneal dialysis [12].

In the current study, the lipid profile was not improved after the intervention. However, the meta-analysis of several randomized controlled trials showed that soy protein and soy isoflavones have positive effects on the blood lipid profile [34,35]. In addition, the replacement of daily protein with soy protein for 8 to 12 weeks results in a reduction in total cholesterol, triglyceride, low-density lipoprotein, and Apo-lipoprotein B 100 (Apo-B 100) [36,37].

One of the limitations in our study was that dietary recall may have potential bias even though the dietitians are skillful in dietary assessment and patients are educated in how to report. The subgroup analysis was not conducted as a small sample size. Despite this, the study shows the value of both dietary advice and oral supplementation. Larger randomized controlled studies are required. In addition, the future study should be conducted in a larger group of patients with a longer follow-up period to examine the long-term effect of oral protein supplementation on the nutritional status and dialysis outcomes.

## 5. Conclusions

Protein intake and serum albumin were significantly increased in all groups with three months of intervention. This indicates that patients’ nutritional status could be gradually improved, as long as their diets improved, whether through nutritional education alone or combined with oral protein supplementation. In addition, milk and soy protein supplements did not significantly change serum phosphorus. This might potentially help patients to control their blood phosphorus level. Health care providers, especially dietitians, should provide dietary consultation and encourage PEW patients to take protein supplements in order to improve their nutritional status. Future longitudinal studies and trials are required for assessing the long-term effects and exploring the mechanism.

## Figures and Tables

**Table 1:** Nutritional ingredients in protein nutritional supplements.

Protein Source	Milk Protein Supplement ^a^	Soy Protein Supplement ^b^
Per package (g)	44.5	48.0
Energy (kcal)	205.0	206.0
Protein (g)	16.8	16.8
Protein (% Energy)	32.9	32.5
Carbohydrate (g)	18.0	19.0
Carbohydrate (% Energy)	35.1	36.9
Fat (g)	7.3	7.0
Fat (% Energy)	32.0	30.6
Saturated Fat (g)	1.4	0.7
Trans Fat (g)	0	0
Ca (mg)	226.8	158.4
PO_4_ (mg)	116.6	211.6
Ca/PO_4_	1.95	0.75

Abbreviations: Ca, calcium; PO_4_, phosphorus; Ca/PO_4_, Ca to PO_4_ ratio. ^a^ S-P93^®^, Sentosa Corporation Limited, Taipei, Taiwan. ^b^ Nu-Reno^®^; Nutritec-enjoy Corporation Limited, Taipei, Taiwan.

**Table 2:** Characteristics of peritoneal dialysis patients at baseline.

Characteristics	Control Group (*n* = 12)	Milk Protein Group (*n* = 21)	Soy Protein Group (*n* = 20)	*p*-Value
Gender, male (%)	4 (33.3)	9 (42.9)	5 (25.0)	0.482
BMI (kg/m^2^)	25.3 ± 6.0	23.1 ± 2.6	23.4 ± 4.1	0.304
Types of PD				
CAPD (%)	5 (41.7)	5 (23.8)	12 (60)	0.063
APD (%)	7 (58.3)	16 (76.2)	8 (40)	0.063
Medical history				
Diabetes (%)	5 (41.7)	4 (19.0)	6 (30)	0.373
Hypertension (%)	9 (75.0)	10 (47.6)	10 (50.0)	0.273
CVD (%)	4 (33.3)	4 (19.0)	6 (30.0)	0.602
CVA (%)	0 (0.0)	0 (0.0)	1 (5.0)	0.431
PAOD (%)	0 (0.0)	1 (4.8)	2 (10.0)	0.483
Urea clearance test				
Total Kt/V_urea_	2.19 ± 0.30	2.11 ± 0.35	2.14 ± 0.41	0.459
Peritoneal Kt/V_urea_	1.87 ± 0.45	1.83 ± 0.53	1.90 ± 0.51	0.599
Residual kidney Kt/V_urea_	0.32 ± 0.34	0.28 ± 0.36	0.24 ± 0.31	0.735
RRF (urine volume > 100 mL/day) (%)	7 (58.3)	11 (52.4)	10 (50.0)	0.900
nPCR (g/kg)	0.94 ± 0.19	1.09 ± 0.22	1.02 ± 0.21	0.163

Abbreviations: BMI, body mass index; PD, peritoneal dialysis; CAPD, continuous ambulatory peritoneal dialysis; APD, automated peritoneal dialysis; CVD, Cardiovascular disease; CVA, Cerebrovascular accident; PAOD, Peripheral Arterial Occlusive Disease; Kt/V_urea_, Urea nitrogen clearance divided by volume of distribution of urea nitrogen; RRF, residual renal function; nPCR, normalized protein catabolic ratio. *p*-value was estimated using one-way ANOVA or Chi-square test, appropriately.

**Table 3:** Daily nutrients’ intake in different groups during intervention.

Nutrients	Control Group (*n* = 12)	Milk Protein Group (*n* = 21)	Soy Protein Group (*n* = 20)
Calorie (kcal/kg)			
Baseline	26.8 ± 8.3	28.6 ± 6.7	27.8 ± 8.4
Month 3	26.1 ± 7.5	30.0 ± 6.2	28.1 ± 6.2
Protein (g/kg)			
Baseline	0.89 ± 0.26	1.10 ± 0.43	1.02 ± 0.32
Month 3	0.97 ± 0.31 *	1.33 ± 0.57 ^a,†^	1.16 ± 0.32 ^a,^*^,†^
Protein (%)			
Baseline	13.5 ± 2.9	15.1 ± 3.5	14.8 ± 3.5
Month 3	14.9 ± 2.5 ^a^	17.7 ± 5.7 ^a^	16.6 ± 3.4 ^a^
Fat (g)			
Baseline	49.2 ± 12.7	52.9 ± 15.6	53.2 ± 24.6
Month 3	51.3 ± 13.8	60.0 ± 18.7 ^a^	55.1 ± 15.7
Fat (%)			
Baseline	28.6 ± 5.4	29.3 ± 5.5	29.1 ± 5.3
Month 3	30.4 ± 6.3 ^a^	31.4 ± 5.3	31.0 ± 7.6
CHO (g)			
Baseline	226.4 ± 31.8	233.0 ± 59.7	225.1 ± 71.3
Month 3	213.7 ± 37.8	221.4 ± 66.5	217.1 ± 66.6
CHO (%)			
Baseline	60.0 ± 6.0	57.7 ± 7.4	57.9 ± 7.4
Month 3	56.8 ± 6.1	52.4 ± 8.3 ^a^	54.0 ± 9.1
Dietary Ca (mg)			
Baseline	260.2 ± 123.7	329.6 ± 141.7	317.4 ± 143.1
Month 3	262.4 ± 134.6 *	487.2 ± 147.3 ^a^	343.3 ± 97.5 *
Dietary PO_4_ (mg)			
Baseline	602.7 ± 104.9	717.5 ± 219.8	693.2 ± 273.8
Month 3	635.8 ± 185.6	928.3 ± 346.4 ^a,^*	810.0 ± 215.3 ^a,^*

Abbreviations: CHO, carbohydrates; Ca, calcium; PO_4_, phosphorus. Data are presented as mean ± standard deviation, the distribution of variables within groups were analyzed by paired *t*-tests (for Calorie, Fat (g), Fat (%), CHO (g), CHO (%), and dietary Ca), and Wilcoxon signed-rank test (for protein (g/kg), protein (%), and dietary PO_4_). The distributions among different groups were analyzed by one-way ANOVA using a Sheffee multiple comparison test (for Calorie, Fat (g), Fat (%), CHO (g), CHO (%), and dietary Ca), and the Mann–Whitney U test (for protein (g/kg), protein (%), and PO_4_). ^a^ *p* < 0.05 indicates the difference between month 3 and baseline. *^,†^ *p* < 0.05 indicates significant difference among different groups.

**Table 4:** Biochemical parameters in different groups during the intervention.

Biochemical	Control Group (*n* = 12)	Milk Protein Group (*n* = 21)	Soy Protein Group (*n* = 20)
Nutritional biomarkers			
BUN (mg/dL)			
Baseline	56.0 ± 10.0	65.0 ± 17.0	66.0 ± 18.0
Month 1	62.0 ± 16.0 *	86.0 ± 23.0 ^†^	78.0 ± 22.0 *^,†^
Month 2	59.0 ± 16.0 *	86.0 ± 21.0 ^†^	78.0 ± 16.0 ^†^
Month 3	61.0 ± 16.0 *	81.0 ± 21.0 ^a,c,†^	79.0 ± 17.0 ^a,†^
Creatinine (mg/dL)			
Baseline	9.6 ± 2.5	11.0 ± 3.5	10.4 ± 2.7
Month 1	10.3 ± 2.9	11.7 ± 4.1	10.6 ± 3.0
Month 2	10.4 ± 2.7	11.6 ± 3.6	10.3 ± 2.7
Month 3	10.4 ± 2.8 ^a^	11.2 ± 3.9	10.8 ± 3.3
Albumin (g/dL)			
Baseline	3.2 ± 0.2	3.2 ± 0.3	3.2 ± 0.3
Month 1	3.4 ± 0.3	3.3 ± 0.3	3.2 ± 0.4
Month 2	3.4 ± 0.3	3.3 ± 0.4	3.3 ± 0.4
Month 3	3.4 ± 0.3 ^a^	3.4 ± 0.3 ^a,b^	3.3 ± 0.4 ^a,b^
Total protein (mg/dL)			
Baseline	6.5 ± 0.6	6.7 ± 0.6	6.4 ± 0.7
Month 1	6.6 ± 0.7	6.9 ± 0.6	6.5 ± 0.8
Month 2	6.6 ± 0.7	6.9 ± 0.6	6.5 ± 0.8
Month 3	6.8 ± 0.7 ^a,b,c^	7.0 ± 0.7 ^a^	6.7 ± 0.7
Hemoglobin (g/dL)			
Baseline	9.6 ± 0.9	10.0 ± 1.4	9.7 ± 1.1
Month 1	9.7 ± 1.3	10.0 ± 1.6	9.9 ± 1.2
Month 2	9.8 ± 1.5	10.0 ± 1.4	9.6 ± 1.5
Month 3	9.8 ± 1.5	10.3 ± 1.4	9.8 ± 1.2
Other biochemical parameters			
Serum Ca (mg/dL)			
Baseline	2.3 ± 0.1	2.4 ± 0.2	2.5 ± 0.3
Month 1	2.2 ± 0.1	2.4 ± 0.3	2.4 ± 0.2
Month 2	2.2 ± 0.2 *	2.4 ± 0.2 *^,†^	2.5 ± 0.3 ^†^
Month 3	2.3 ± 0.1 *	2.4 ± 0.2 *^,†^	2.5 ± 0.2 ^†^
Serum PO_4_ (mg/dL)			
Baseline	4.2 ± 1.2	5.1 ± 1.3	5.0 ± 1.7
Month 1	4.6 ± 0.9	5.3 ± 1.5	5.3 ± 1.4
Month 2	4.8 ± 0.9	5.2 ± 1.3	5.1 ± 1.4
Month 3	5.3 ± 1.4 ^a^	5.5 ± 1.8	5.2 ± 1.3
Serum Na (mEq/L)			
Baseline	132.0 ± 4.0	134.0 ± 4.0	134.0 ± 4.3
Month 1	133.0 ± 3.0	133.0 ± 5.0	134.0 ± 4.4
Month 2	133.0 ± 4.0	133.0 ± 4.0	132.0 ± 4.0
Month 3	133.0 ± 3.0	132.0 ± 4.0 *^,†^	132.0 ± 3.2 *^,†^
Serum K (mEq/L)			
Baseline	3.4 ± 0.8	3.7 ± 0.5	3.7 ± 0.6
Month 1	3.5 ± 0.7	3.6 ± 0.5	3.7 ± 0.7
Month 2	3.6 ± 1.0	3.5 ± 0.5*	3.6 ± 0.8
Month 3	3.7 ± 0.9	3.7 ± 0.5 ^a^	3.8 ± 0.7
TC (mg/dL)			
Baseline	181.0 ± 47.0	174.0 ± 41.0	172.0 ± 32.0
Month 3	180.0 ± 40.0	174.0 ± 43.0	181.0 ± 42.0
TG (mg/dL)			
Baseline	235.0 ± 311.0	142.0 ± 96.0	124.0 ± 65.0
Month 3	235.0 ± 291.0	125.0 ± 73.0	117.0 ± 57.0

Abbreviations: BUN, blood urea nitrogen; Ca, calcium; K, potassium; Na, sodium; PO_4_, phosphorous; TC, total cholesterol; TG, triglyceride. Data are presented as mean ± standard deviation, the distribution of variables within groups were analyzed by one-way repeated measured ANOVA and Fisher’s least significant difference test (BUN, creatinine, total protein, hemoglobin, Ca, Na, PO_4_), by the Wilcoxon signed-rank test (for Albumin, K, TC, and TG). Distribution of variables among different groups was analyzed by one-way ANOVA and Sheffee multiple comparison analysis tests (BUN, creatinine, total protein, hemoglobin, Ca, Na, PO_4_), by Mann–Whitney U test (for Albumin, K, TC, and TG). ^a^ *p* < 0.05 indicates the difference between month 3 and baseline. ^b^ *p* < 0.05 indicates the difference between month 3 and month 1. ^c^ *p* < 0.05 indicates the difference between month 3 and month 2. *^,†^ *p* < 0.05 indicates a significant difference among different groups.

## Supplementary Materials

The following are available online at https://www.mdpi.com/2227-9032/7/4/135/s1, Table S1: Consort 2010 checklist of information to include when reporting a randomised trial.
